# The Auditory Steady-State Response and the Relationship between Electrophysiological and Behavioural Thresholds

**DOI:** 10.3390/diagnostics14151617

**Published:** 2024-07-26

**Authors:** Cyntia Barbosa Laureano Luiz, Daniela Gil, Piotr Henryk Skarzynski, Magdalena Beata Skarżyńska, Milaine Dominici Sanfins, Marisa Frasson de Azevedo

**Affiliations:** 1 Department of Speech-Hearing-Language, Universidade Federal de São Paulo, São Paulo 04044-020, Brazil; cyntialuiz@yahoo.com.br (C.B.L.L.); dgil@unifesp.br (D.G.); marisa.frasson@uol.com.br (M.F.d.A.); 2 Post-Graduate Program in Clinical Audiology, Instituto de Ensino e Pesquisa Albert Einstein, São Paulo 05652-000, Brazil; 3 Clinic of Audiology, Universidade Federal de São Paulo, São Paulo 04044-020, Brazil; 4 Department of Teleaudiology and Screening, World Hearing Center, Institute of Physiology and Pathology of Hearing, 05-830 Kajetany, Poland; p.skarzynski@csim.pl; 5 ENT Department, Maria Curie-Skłodowska University, 20-031 Lublin, Poland; 6 Center of Hearing and Speech Medincus, 05-830 Kajetany, Poland; 7 Department of Otolaryngology, Institute of Sensory Organs, 05-830 Warsaw, Poland; 8 Heart Failure and Cardiac Rehabilitation Department, Medical University of Warsaw, 02-091 Warsaw, Poland; 9 World Hearing Center, 05-830 Kajetany, Poland; 10 Department of Pharmacotherapy and Pharmaceutical Care, Pharmaceutical Department, Medical University of Warsaw, 02-091 Warsaw, Poland; m.skarzynska@csim.pl; 11 Institute of Sensory Organs, 05-830 Kajetany, Poland; 12 Center of Hearing and Speech, 05-830 Nadarzyn, Poland

**Keywords:** electrophysiology, auditory evoked potentials, hearing loss, audiometry, pure tone audiometry, hearing tests

## Abstract

Background: This study examined the relationship between behavioural thresholds as measured by pure tone audiometry and electrophysiological thresholds measured by the Auditory Steady-State Response (ASSR) in children with normal hearing and sensorineural hearing loss. Materials and Methods: After being assessed, 45 children of both sexes, ranging in age from 5 to 15, were split into four groups: 10 with moderate to moderately severe sensorineural hearing loss (G2M); 10 with steeply sloping sensorineural hearing loss (G2D); 10 with profound and severe sensorineural hearing loss (G2S); and 15 with normal hearing (G1). ASSR, tympanometry, acoustic reflex testing, pure tone audiometry, and speech audiometry (SRT and SDT) were performed. Results: The electrophysiological maximum in the group with normal hearing thresholds varied from 19 to 27 dB NA. The correlation in the group with moderate to moderately severe hearing loss was 0.42–0.74. The correlation in the steeply sloping hearing loss group was 0.68–0.94. The correlation in the group of people with profound and severe hearing loss was 0.59–0.86. The normal hearing group’s mean differences in ASSR threshold and audiometric threshold ranged from −0.3 to 12 dB, in the moderate and moderately severe hearing loss group from −9 to 2 dB, in the steeply sloping hearing loss group from 1.4 to 7.5 dB, and in the severe and profound hearing loss group from −0.40 to 8.5 dB. Conclusion: As expected, there was no strong relationship between behavioural and electrophysiological thresholds in the group with normal hearing. But in children with hearing loss, there was a strong correlation between electrophysiological and behavioural thresholds; this relationship was especially evident in children with severe and profound hearing loss and those with steeply sloping hearing loss.

## 1. Background

Neonatal hearing screening brings a great challenge to audiologists to establish the degree of hearing loss and the audiometric configuration in newborns. To achieve the objective of making a diagnosis accurately and objectively, behavioural and electrophysiological procedures need to be included in hearing assessment protocols for children. Therefore, the Auditory Steady-State Response (ASSR) based on evoked potentials has been recommended to complement a child’s audiological assessment. The ASSR is a periodic electrophysiological response, which occurs as a result of an auditory stimulus presented at a frequency rate fast enough to cause overlapping responses to successive stimuli. The continuous neural response of auditory neurons in the brain stem is called the steady state [1,2].

Electrophysiological assessment has the advantage of not depending on the subject’s collaboration to obtain a response, in addition to allowing evaluation with stimuli of specific frequencies. It can be recorded through the auditory brainstem response (ABR) with tone bursts (TBs) or through the response obtained from the Auditory Steady-State Response (ASSR). The stimuli used in electrophysiological assessment involve certain frequencies in the cochlea, such as click stimuli that activate high-frequency areas around 2000 Hz to 4000 Hz, being a comprehensive frequency range, which stimulates a wide portion of the basilar membrane in the cochlea. Tone burst and ASSR stimuli activate low and high frequencies in the cochlea, with the most commonly used in audiological clinics being 500 Hz, 1000 Hz, 2000 Hz, and 4000 Hz.

The advantages of ASSR in relation to ABR-TBs are (a) shorter completion time as the multifrequency and dichotic presentation provides faster recording; (b) obtaining higher sound pressure level thresholds when compared to ABR-click and ABR-TBs, as it presents higher maximum output values; and (c) objective analysis: the ASSR analysis, with automatic detection of responses, enables the reduction in risks arising from the evaluator’s subjective assessment [1,2].

The literature has emphasized the use of ASSR to predict hearing thresholds in children with different degrees of hearing loss, with this method being considered a complementary instrument in a child’s audiological assessment [3,4,5,6,7,8,9,10,11].

However, the ASSR for predicting behavioural hearing thresholds has not yet been systematically included in clinical routines, although some studies have already identified a good correlation between electrophysiological and behavioural thresholds, especially in individuals with severe and profound hearing loss [10,12,13,14,15,16].

As ASSR is a relatively recent procedure in our clinical routines, this study was prompted by the need to carry out more studies in children on the correlations between thresholds obtained by this procedure and behavioural auditory thresholds as defined through pure tone audiometry. In most studies, the selected sample has been composed of adults, and there have been few studies on children.

Thus, this study aimed to verify the correlation between the electrophysiological thresholds obtained by the Auditory Steady-State Response (ASSR) and the behavioural thresholds obtained by pure tone audiometry in children with normal auditory thresholds and those with sensorineural hearing losses of different degrees.

## 2. Materials and Methods

This cross-sectional observational analytical study was approved by the Research Ethics Committee of the Universitidade Federal de São Paulo (São Paulo, Brazil) (n° 0669/11). All parents of the children were informed about the procedures to be carried out and signed an informed consent form before participating in this study; older children were also informed about the procedures to be carried out and signed the consent form. The final sample consisted of 45 children of both sexes aged between 5 and 15 years old, which were divided into the following two groups:

1. Control Group (G1): 15 children with hearing thresholds within normal limits.
2. Study Group (G2): composed of 30 children with sensorineural hearing loss, of whom 10 had moderate and moderately severe hearing loss (G2M), 10 had severe to profound hearing loss (G2S), and 10 had descending hearing loss (G2D).

The inclusion criteria were age between 5 and 15 years, type A tympanometric curves bilaterally [17], and consistent responses to pure tone audiometry. Individuals with conductive and neurological changes were excluded. All individuals underwent anamnesis, pure tone audiometry, acoustic immittance measurements, and electrophysiological assessment of hearing (ASSR). All procedures were performed by the same evaluator.

Tonal and speech audiometry was performed in a soundproof booth with a model MA-41 audiometer and TDH-39 supra-aural headphones (Maico Diagnostics^®^, Berlin, Germany). Hearing thresholds were determined at frequencies from 0.25 to 8 kHz and individuals were instructed to raise their hand when they heard a stimulus, even if it was at low intensity. Thresholds were investigated using the descending (10 dB) and ascending (5 dB) techniques. The lowest level at which the patient responded to 50% of the sound presentations was considered the auditory threshold.

The degree of hearing loss was classified by the average thresholds at 0.5, 1, and 2 kHz in pure tone audiometry [18]. Hearing was considered normal if the average was below 25 dBHL. Hearing loss was classified as moderate or moderately severe when the average was 41–70 dBHL, and severe and profound hearing loss when the average was greater than 71 dBHL. The audiogram configuration was classified as horizontal when there was a difference of no more than 5 dB between the thresholds of different frequencies and descending when there was a difference of 5–20 dB per octave [19].

Measurements of acoustic immittance, tympanometry, and acoustic reflexes were carried out using AT-235 equipment (Interacoustics^®^, Middelfart, Denmark). Individuals were instructed to remain quiet, without moving or speaking. A tympanometric curve was considered type A when the maximum compliance was between +100 and −100 daPa and the middle ear volume was between 0.3 and 1.6 mL [17].

ASSRs were performed with the Smart EP equipment (Intelligent Hearing Systems^®^, Miami, FL, USA). The examination was carried out in an acoustically and electrically treated room. The children sat comfortably in a reclining chair and were instructed to remain still to avoid myogenic artefacts, especially movements of the head and neck. Before the start of the test, the subject’s skin was cleaned with abrasive paste and the electrodes were positioned so that the recording was carried out ipsilateral to the stimulated ear. Impedances were maintained <5 kΩ, and the electrodes were arranged with M1 (−), Fz (+), and M2 (ground). Acoustic stimuli were presented through ER-3B insert headphones, adapted to the external auditory canal using disposable foam plugs. The examination was performed without the use of sedation. Stimulation was monaural, and the presentation of the stimuli was mixed (multifrequency at the beginning of the exam and a single frequency near threshold). ASSRs were evoked using the descending (10 dB) and ascending (5 dB) techniques, with the maximum output of the equipment being 117 dB SPL. Electrophysiological thresholds were obtained in dB SPL and converted to dB NA by the equipment itself. The corrections were as follows: −26 dB for 0.5 kHz, −11 dB for 1 kHz, −13 dB for 2 kHz, and −19 dB for 4 kHz.

ASSRs were detected automatically by comparing the signal amplitude and the noise amplitude at the presentation rate. These responses were divided into signal and noise, using the *F*-statistic. The response was considered present when the signal-to-noise ratio was ≥6.13 dB, with a response greater than 0.0125 μV, electrical noise lower than 0.05 μV, and residual noise below 0.70 μV. Statistical analysis was performed every 20 scans, using the maximum presentation of 400 scans, with a 30–300 Hz filter. The criterion used to interrupt the recording was the presence or absence of a response when residual noise <0.70 μV (as suggested in the equipment manual). In cases where the noise did not reach this limit after 400 scans, the exam was redone. The stimuli used were tone pips, modulated at 100% amplitude with carrier frequencies from 500 to 4000 Hz at modulation frequencies of 79, 87, 95, and 103 Hz in the right ear and 77, 85, 93, and 101 Hz in the left ear, respectively.

Although different transducers were used to obtain behavioural thresholds (supra-aural headphones TDH-39) and electrophysiological thresholds (ER-3B insert phones), the thresholds for the insert headphones were not corrected in behavioural audiometry since the correction factors for frequencies from 0.5 to 4 kHz are only 0–2 dB [20] (and 2 dB has no clinical validity since thresholds were determined in 5 dB steps).

For statistical analysis, descriptive analyses were performed (mean, standard deviation, median, minimum, and maximum) for both electrophysiological and behavioural thresholds in each group, and Pearson and Spearman linear correlation tests were used to assess correlations between the two thresholds.

## 3. Results

### 3.1. Comparison between Electrophysiological and Behavioural Thresholds in the Total Population

Table 1 presents the correlations between the behavioural thresholds obtained by pure tone audiometry and the electrophysiological thresholds obtained by ASSR, at frequencies from 0.5 to 4 kHz in the 45 individuals. All correlations were greater than 0.89, indicating a strong correlation between behavioural and electrophysiological thresholds.

**Figure 1:**
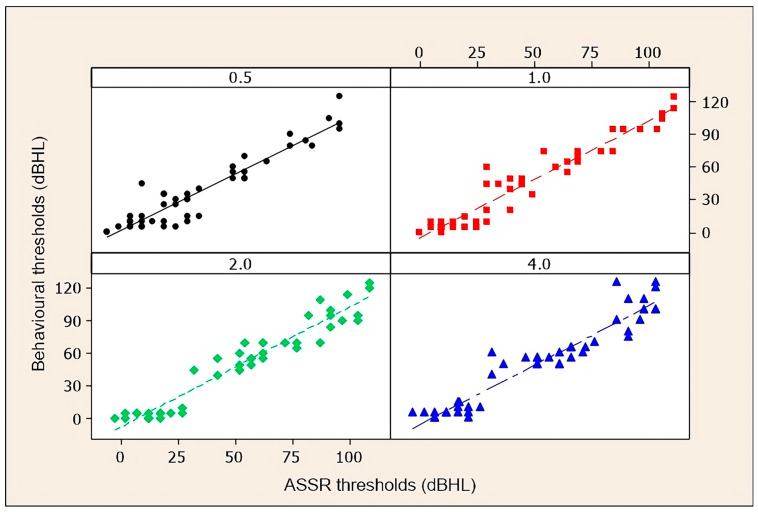
Scatter diagrams for the right ear of behavioural thresholds (and electrophysiological thresholds at 0.5, 1, 2, and 4 kHz in the total sample).

**Figure 2:**
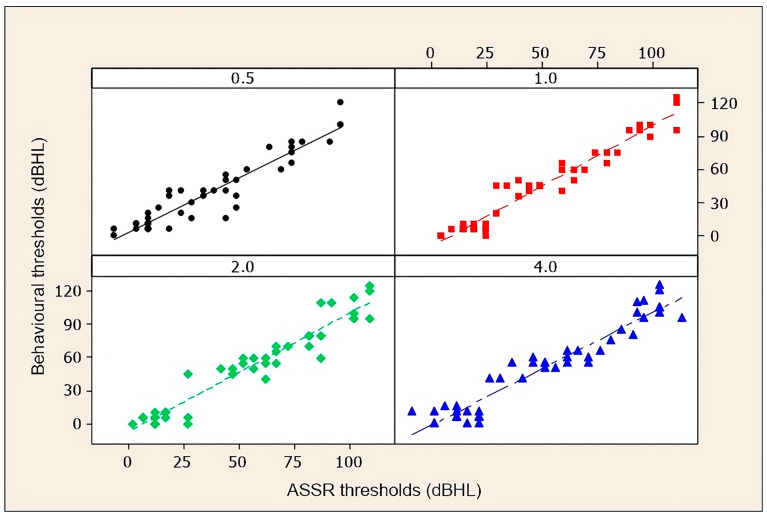
Scatter diagrams for the left ear of behavioural thresholds and ASSR thresholds at 0.5, 1, 2, and 4 kHz in the total sample.

Figure 1 and Figure 2 are scatter diagrams of the behavioural auditory thresholds obtained by tonal audiometry and the electrophysiological thresholds obtained by ASSR, at 0.5, 1, 2, and 4 kHz in the total sample (45 individuals).

The average differences between behavioural and electrophysiological auditory thresholds in the total population are presented in Table 2.

### 3.2. Comparison between Behavioural and Electrophysiological Thresholds in Groups with Normal Hearing and Different Degrees of Hearing Loss

**Figure 3:**
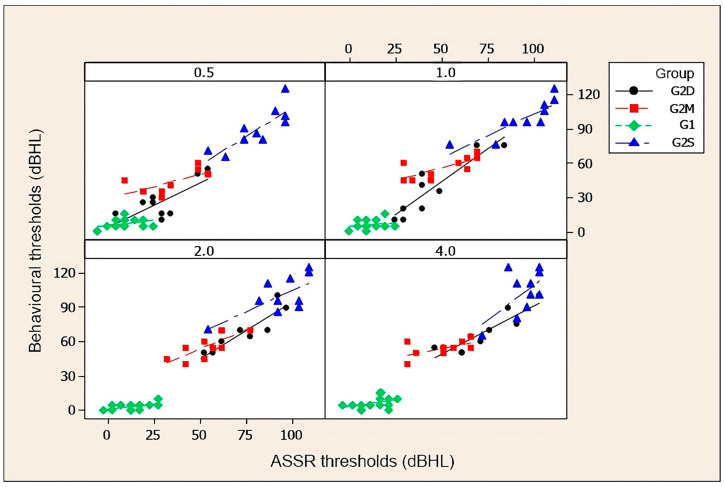
Scatter diagrams for right ears of behavioural auditory thresholds and electrophysiological thresholds at 0.5, 1, 2, and 4 kHz. Key: G1, control group; G2M, study group with moderate and moderately severe sensorineural hearing loss; G2S, study group with severe to profound sensorineural hearing loss; and G2D, study group with descending sensorineural hearing loss.

**Figure 4:**
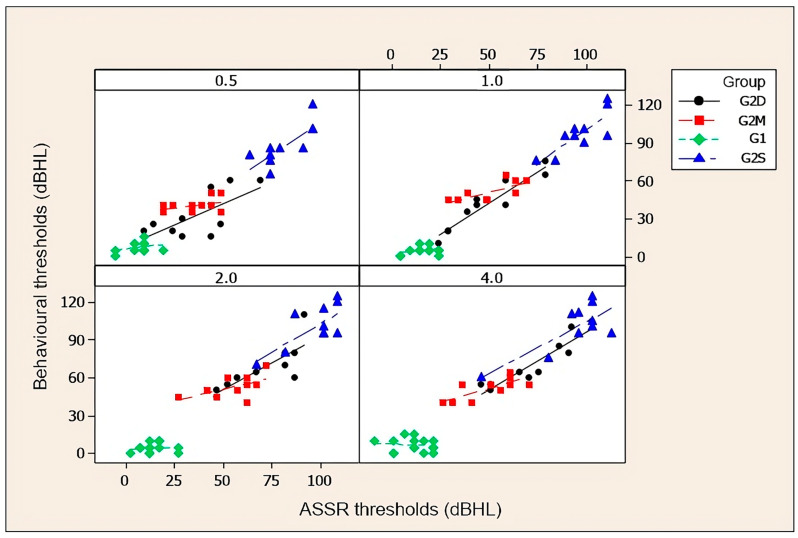
Scatter diagrams for left ears of behavioural auditory thresholds and electrophysiological thresholds at 0.5, 1, 2, and 4 kHz. Key: G1, control group; G2M, study group with moderate and moderately severe sensorineural hearing loss; G2S, study group with severe to profound sensorineural hearing loss; and G2D, study group with descending sensorineural hearing loss.

Figure 3 and Figure 4, and the corresponding Table 3 and Table 4, show correlations between behavioural and electrophysiological auditory thresholds at 0.5, 1, 2, and 4 kHz obtained in groups with auditory thresholds within normal limits (G1), with sensorineural hearing loss of moderate and moderately severe degrees (G2M), with severe to profound sensorineural hearing loss (G2S), and descending sensorineural hearing loss (G2D). Pearson’s correlation coefficient was used for the group with normal hearing thresholds and Spearman’s was used for the others.

The average differences between the electrophysiological and behavioural auditory thresholds in the different groups are presented in Table 5 (right ear) and Table 6 (left ear).

## 4. Discussion

The results of this study demonstrate the applicability of ASSR in audiological diagnosis, finding good correlations with pure tone audiometry. Therefore, the ASSR can be considered an important tool for predicting the degree and configuration of the audiogram, which can be useful in selecting and adapting hearing aids, especially in young children.

The results of the present study demonstrate that, in 45 children, there was a strong correlation between electrophysiological and behavioural thresholds. A strong correlation between the two tests has previously been described in adults. There have been few studies carried out on children and adolescents. One study involving children, adolescents, and adults observed correlations (in frequencies from 0.5 to 4 kHz) of 0.72, 0.70, 0.76, and 0.91, respectively [21], lower values than in the present study and similar to another study on adolescents and adults with hearing loss [22]. One other study evaluating children, adolescents, and adults did see correlations higher than 0.80, similar to the present work [13,14,15,16,17,18,19,20,21,22,23].

We found median differences of 0.31 and 5.36 dB (Table 2) between electrophysiological and behavioural thresholds, values which agree with those in the literature [4,6,10,13,24,25,26]. Some other studies have found higher differences [8,9,11,27,28,29,30]. The literature shows the biggest differences at frequencies of 1 and 2 kHz and the smallest at 4 kHz. In general, the smaller differences are seen at higher frequencies [31].

The correlation between electrophysiological and behavioural thresholds varied depending on the group. In the group with normal thresholds, there was no correlation between electrophysiological and behavioural thresholds. These findings agree with the majority of studies in the literature [12,13,14,30]. In the group with moderate to moderately severe loss, the correlation was 0.42 to 0.74. These findings agree with some studies that have studied the correlation in individuals with moderate and moderately severe loss at frequencies from 0.5 to 4 kHz [21,22,26]. However, other studies have found higher correlations (0.67 and 0.93) in populations with moderate and moderately severe hearing loss [13].

In the present study, the best correlations were obtained in the group with descending sensorineural hearing loss (0.68 and 0.94) and with severe and profound sensorineural hearing loss (0.59 and 0.86).

Other studies have demonstrated a strong correlation between electrophysiological and behavioural thresholds in cases with descending hearing losses, such as a study on workers exposed to noise [28]. Another study carried out with children who had descending losses found a strong correlation between 0.5 and 2 kHz, but only a moderate correlation at 4 kHz [32]. In our group with descending hearing loss, the worst correlation was observed at a frequency of 0.5 kHz, similar to the literature. One factor responsible for poor correlation at 0.5 kHz is cochlear tonotopy, which provides greater sound dispersion, resulting in a decrease in the amplitude of the response at this frequency, which is represented at the apical end of the cochlea [21]. Poorer responses at 0.5 kHz can be explained by the interference of electrophysiological and/or environmental noise at low frequencies.

One study in individuals with severe to profound horizontal sensorineural hearing loss found a strong correlation (*r* = 0.91) between electrophysiological and behavioural thresholds in individuals aged 5 and 74 years [12]. Another study on individuals aged 10 to 15 years with severe and profound losses found a strong correlation only at 1 kHz, with moderate correlations at other frequencies [6].

We found the largest mean differences (−0.3 and 12 dB NA) between electrophysiological and behavioural thresholds in the group with normal thresholds. In this group, electrophysiological thresholds were worse than behavioural thresholds. For this reason, the use of correction factors is recommended, with greater corrections at low frequencies.

The extended distance between the sites of generation and the surface electrodes that capture the response (the far-field potential) is one of the main reasons for the differences, for the distance means that smaller responses have to be extracted from the background noise.

The strong correlations between the electrophysiological and behavioural thresholds [33,34,35] obtained in the present study demonstrate the usefulness of the ASSR in the early diagnosis of hearing loss, especially for severe and profound sensorineural losses with a horizontal configuration and in cases of descending sensorineural hearing losses. In this way, children who do not yet respond to pure tone audiometry, the gold standard in audiology, can benefit from the use of ASSR, which provides good reliability in determining thresholds and can contribute effectively to the fitting of hearing aids during the first few months of life.

## 5. Conclusions

A strong correlation was found between electrophysiological and behavioural thresholds in the total population of 45 children, with the relationship being stronger in the groups with descending hearing loss and with severe and profound hearing loss.

## Figures and Tables

**Table 1:** Spearman’s correlation coefficient (*R*) between the electrophysiological threshold (dB NA) and behavioural auditory threshold (dB HL) by frequency and ear (without distinction of degree of loss).

Frequency kHz	Right Ear			Left Ear		
		*R*	*p*-Value		*R*	*p*-Value
0.5	Threshold = 1.42 ± 1.05	0.893	<0.001 *	Threshold = 1.61 ± 1.00	0.919	<0.001 *
1	Threshold = −4.55 ± 1.07	0.945	<0.001 *	Threshold = −10.1 ± 1.10	0.952	<0.001 *
2	Threshold = −7.66 ± 1.10	0.953	<0.001 *	Threshold = −7.55 ± 1.08	0.944	<0.001 *
4	Threshold = −4.66 ± 1.08	0.946	<0.001 *	Threshold = −1.05 ± 1.02	0.934	<0.001 *

Legend: *p*-value, * *p* ≤ 0.05.

**Table 2:** Mean differences between electrophysiological and behavioural thresholds in the total population.

Ear	Frequency (kHz)	*N*	Mean	SD	Minimum	Median	Maximum
Right	0.5	45	−3.00	11.00	–36	–4	19
	1	45	1.33	10.82	–31	1	19
	2	45	2.16	10.88	–23	2	22
	4	45	0.31	12.18	–39	1	21
Left	0.5	45	−1.60	10.61	–24	–1	29
	1	45	5.36	9.48	–16	4	24
	2	45	3.24	11.07	–23	2	27
	4	45	−0.11	11.03	–22	1	21

**Table 3:** Right ear correlation coefficients between the electrophysiological threshold (dB HL) and behavioural auditory threshold (dB HL) by group.

Right Ear								
	G1		G2M		G2S		G2D	
Frequency (kHz)	*R*	*p*-Value	*R*	*p*-Value	*R*	*p*-Value	*R*	*p*-Value
0.5	0.424	0.116	0.689	0.028 *	0.823	0.003 *	0.730	0.016 *
1	0.189	0.500	0.733	0.016 *	0.868	0.001 *	0.905	<0.001 *
2	0.434	0.106	0.711	0.009 *	0.715	0.020 *	0.926	<0.001 *
4	0.422	0.116	0.556	**0.095 #**	0.594	**0.070 #**	0.893	0.001 *

Key: G1, control group; G2M, study group with moderate and moderately severe sensorineural hearing loss; G2S, study group with severe to profound sensorineural hearing loss; and G2D, study group with descending sensorineural hearing loss. * (*p* < 0.05). #: the tendency toward a significant difference.

**Table 4:** Left ear correlation coefficients between the electrophysiological threshold (dB HL) and behavioural auditory threshold (dB HL) by group.

Left Ear								
	G1		G2M		G2S		G2D	
Frequency (kHz)	*R*	*p*-Value	*R*	*p*-Value	*R*	*p*-Value	*R*	*p*-Value
0.5	0.424	0.116	0.424	0.222	0.779	**0.080 #**	0.687	0.028 *
1	0.189	0.500	0.699	0.025 *	0.823	0.003 *	0.948	<0.001 *
2	0.434	0.106	0.547	0.102	0.734	0.016 *	0.801	0.005 *
4	0.422	0.116	0.747	0.013 *	0.776	**0.080 #**	0.929	<0.001 *

Key as per Table 3. * (*p* < 0.05). #: the tendency toward a significant difference.

**Table 5:** Mean differences for right ears between electrophysiological and behavioural thresholds in the different groups.

Right Ear								
	G1		G2M		G2S		G2D	
Frequency (kHz)	Mean	SD	Mean	SD	Mean	SD	Mean	SD
0.5	1.00	7.97	−9.0	11.83	−8.50	10.07	2.5	10.81
1	6.33	7.53	−5.5	11.41	−4.50	8.96	6.5	10.61
2	9.33	7.76	−2.5	8.32	−6.80	12.73	5.0	6.75
4	6.40	7.85	−4.0	11.06	−8.40	16.03	4.2	8.18

Key as per Table 3.

**Table 6:** Mean differences for left ears between electrophysiological and behavioural thresholds in the different groups.

			Left Ear					
	G1		G2M		G2S		G2D	
Frequency (kHz)	Mean	SD	Mean	SD	Mean	SD	Mean	SD
0.5	−0.33	6.23	−5.0	10.49	−5.70	9.72	4.0	14.72
1	11.67	6.51	−0.5	9.85	−0.40	9.24	7.5	6.69
2	9	7.51	2.0	11.30	−3.40	11.88	2.5	11.65
4	4	11.15	−2.0	10.33	−5.90	12.95	1.4	7.43

Key as per Table 3.

## Data Availability

The raw data supporting the conclusions of this article will be made available by the authors on request due to privacy concerns. The data in tables are deposited in the UNIFESP research data repository and can be accessed via the link: http://repositorio.unifesp.br/handle/11600/22542.
